# Elevation of Trimethylamine-N-Oxide in Chronic Kidney Disease: Contribution of Decreased Glomerular Filtration Rate

**DOI:** 10.3390/toxins11110635

**Published:** 2019-11-01

**Authors:** Caroline C. Pelletier, Mikael Croyal, Lavinia Ene, Audrey Aguesse, Stephanie Billon-Crossouard, Michel Krempf, Sandrine Lemoine, Fitsum Guebre-Egziabher, Laurent Juillard, Christophe O. Soulage

**Affiliations:** 1 Hospices Civils de Lyon, Service de Néphrologie, Dialyse et Hypertension Artérielle, Hôpital E Herriot, F-69003 Lyon, France; caroline.pelletier02@chu-lyon.fr (C.C.P.); raluca@gmail.com (L.E.); sandrine.lemoine01@chu-lyon.fr (S.L.); fitsum.guebre-egziabher@chu-lyon.fr (F.G.-E.); laurent.juillard@univ-lyon1.fr (L.J.); 2 Université de Lyon, INSERM U1060, CarMeN, INSA de Lyon, Univ Lyon-1, F-69621 Villeurbanne, France; 3 NUN, INRA, CHU Nantes, UMR 1280, PhAN, IMAD, CRNH-O, F-44000 Nantes, France; mikael.croyal@univ-nantes.fr (M.C.); Audrey.Aguesse@univ-nantes.fr (A.A.); stephanie.crossouard@univ-nantes.fr (S.B.-C.); Michel.Krempf@univ-nantes.fr (M.K.); 4 CRNH-O Mass Spectrometry Core Facility, F-44000 Nantes, France; 5 ELSAN, clinique Bretéché, F-44000 Nantes, France

**Keywords:** uremic toxin, Trimethylamine-N-oxide, renal clearance, chronic kidney disease, hemodialysis

## Abstract

Gut microbiota-dependent Trimethylamine-N-oxide (TMAO) has been reported to be strongly linked to renal function and to increased cardiovascular events in the general population and in Chronic Kidney Disease (CKD) patients. Considering the lack of data assessing renal handling of TMAO, we conducted this study to explore renal excretion and mechanisms of accumulation of TMAO during CKD. We prospectively measured glomerular filtration rate (mGFR) with gold standard methods and plasma concentrations of trimethylamine (TMA), TMAO, choline, betaine, and carnitine by LC-MS/MS in 124 controls, CKD, and hemodialysis (HD) patients. Renal clearance of each metabolite was assessed in a sub-group of 32 patients. Plasma TMAO was inversely correlated with mGFR (*r^2^* = 0.388, *p* < 0.001), confirming elevation of TMAO plasma levels in CKD. TMAO clearances were not significantly different from mGFR, with a mean ± SD TMAO fractional excretion of 105% ± 32%. This suggests a complete renal excretion of TMAO by glomerular filtration with a negligible participation of tubular secretion or reabsorption, during all stages of CKD. Moreover, TMAO was effectively removed within 4 h of hemodiafiltration, showing a higher fractional reduction value than that of urea (84.9% ± 6.5% vs. 79.2% ± 5.7%, *p* = 0.04). This study reports a strong correlation between plasma TMAO levels and mGFR, in CKD, that can be mainly related to a decrease in TMAO glomerular filtration. Clearance data did not support a significant role for tubular secretion in TMAO renal elimination.

## 1. Introduction

Trimethylamine-N-oxide (TMAO) is a small amine plasma compound of 75.1 Da derived from intestinal microbial metabolism. Gut flora produces trimethylamine (TMA) either from foods containing TMA or TMAO, or from diet precursors that are identified as choline, phosphatidylcholine, betaine, and carnitine [1,2,3]. TMA is then actively absorbed through the intestinal barrier into the bloodstream, N-oxidized by the hepatic enzyme flavin-containing monooxygenase isoform 3 (FMO3) in humans [4], and excreted as TMAO in urine [5,6]. TMAO has no known specific function in human body but a deficit in FMO3 has been shown to lead to a rare pathological status named trimethylaminuria [7]. This disease is characterized by an increased TMA concentration in all body fluids and was initially termed fish malodor syndrome in regard to its main symptom of unpleasant fish smell. However, lack of TMAO has not been reported to be associated with any pathological condition.

Conversely, over the last two decades, several large clinical studies have shown that plasma TMAO levels associate with cardiovascular risk [8,9,10,11], suggesting a proatherogenic role for TMAO. First, Wang et al. identified an increased level of TMAO in 50 patients with high cardiovascular morbi-mortality and confirmed the correlation between higher levels of TMAO and cardiovascular diseases in a cohort of 1876 patients [10]. The same group published three more consistent studies confirming the strong link between increased TMAO and cardiovascular outcomes, and showing a key role of gut microbial metabolism of phosphalidylcholine [9], choline, betaine [11], and carnitine [8] in the generation of TMAO. Patients with Chronic Kidney Disease (CKD) are also characterized by an increased cardiovascular risk that is not fully explained by traditional risk factors [12]. Furthermore, previous studies have already reported that TMAO accumulates in CKD patients [5,13]. These results were confirmed by clinical studies. In 2006, Bain et al. showed that the concentrations of TMA and TMAO in pre-dialysis plasma were significantly higher than the corresponding levels in healthy subjects [13]. In addition, Stubbs et al. described a strong inverse association of serum TMAO concentration with estimated glomerular filtration rate (eGFR) [14]. The authors suggested that this was the consequence of decreased GFR rather than tubular dysfunction. So far, only one study used measured GFR to determine the association between plasma TMAO and CKD, but the authors concentrated on CKD stages 3 to 5 [15]. Moreover, some studies have reported that the elevation of TMAO in CKD is associated with an increased risk of mortality [15,16,17]. The effects of hemodialysis (HD) on TMAO plasma levels and removal also remain unclear. Although a 2.5 to 40-fold increase in TMAO levels has been reported in HD patients when compared to control subjects with normal kidney function [14,15,18], it is unsure whether elevation of TMAO is really associated with higher cardiovascular risk in this population [19].

Although numerous studies have reported increased TMAO levels in CKD, data assessing renal metabolism (filtration and excretion) of TMAO is still lacking. We therefore conducted a prospective study to address this question. More specifically, we aimed to determine TMA, TMAO, choline, betaine, and carnitine concentrations in all stages of CKD, using gold standard measured glomerular filtration rate (mGFR) and renal clearance measures in order to assess the role of decreased GFR in TMAO increase. Moreover, we measured concentrations of these solutes before and after hemodialysis treatment to assess the effect of hemodialysis on their removal.

## 2. Results

### 2.1. Patient Characteristics and Biochemical Parameters

**Figure 1:**
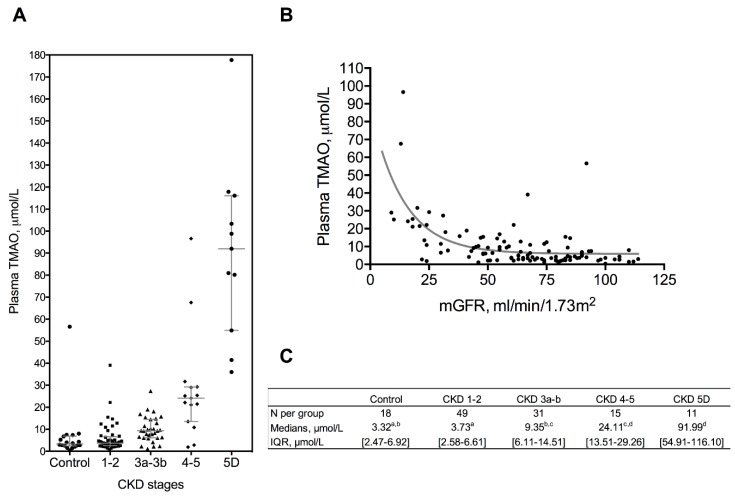
Plasma TMAO levels according to CKD stages. (**A**–**C**): plasma TMAO is increased in chronic kidney disease. Data for plasma TMAO are shown according to CKD stages and are expressed as medians [interquartile range]. Groups were compared with a Kruskal–Wallis test. Different letters indicate significant differences (*p* < 0.05) between groups. (**B**) Plasma TMAO concentrations is negatively correlated with mGFR measured by gold standard method (*r^2^* = 0.388).

A total of 124 patients, including 11 hemodialysis patients, were recruited in our study. The main characteristics of the patients are shown in Table 1. GFR was measured for non-dialysis participants

and these were separated into four groups: controls (*n* = 18), CKD stages 1–2 (*n* = 49), CKD stages 3a–3b (*n* = 31), and CKD stages 4–5 (*n* = 15). There were significant differences between groups in sex ratio, age, and data concerning renal function, plasma bicarbonate, proteins, triglycerides, total cholesterol, LDL-cholesterol, and proportion of lipid lowering treatments. Plasma TMAO concentration was significantly increased in patients with mGFR < 60 mL/min.1.73 m^2^ according to the CKD stages, with an increase of median concentration by 3 (CKD 3a–b), 7 (CKD 4–5), and 28 (CKD 5D) fold compared to controls (Figure 1A–C). mGFR was negatively correlated to TMAO plasma concentration (*r^2^* = 0.388, *p* < 0.0001) (Figure 1B). In univariate analysis, plasma TMAO was positively correlated with age, urinary albumin/creatinine ratio, and uric acid; and negatively correlated with bicarbonates (Table 2).

No correlation was found between plasma TMAO and BMI, triglycerides, LDL-cholesterol, HDL-cholesterol, or estimated protein intake. Plasma TMAO was not correlated with gender with a median [interquartile range, IQR] concentration of 6.98 µmol/L [3.08–14.40] in males and 4.60 µmol/L [2.82–10.01] in females (*p* = 0.168). Plasma concentrations of choline, betaine, carnitine, and TMA for all groups and according to CKD subgroups, are shown in Table 3. As for TMAO, choline and carnitine were inversely correlated with mGFR in CKD patients (*r_s_* = −0.26, *p* = 0.0059 and *r_s_* = −0.32, *p* = 0.0005 respectively). In addition, hemodialysis participants exhibited significantly higher concentrations of choline and significantly lower concentration of carnitine than all CKD participants (*p* < 0.0001).

TMA was positively correlated to renal function (*r_s_* = 0.37, *p* < 0.0001), whereas there was no relationship between betaine and mGFR. Among precursors of TMAO, only carnitine was weakly correlated with plasma concentrations of TMAO (*r_s_* = 0.20, *p* = 0.035).

### 2.2. Renal Excretion of TMAO and Precursors

Among participants, a subgroup of 32 patients (five controls, 19 CKD stage 1–2, and eight CKD stage 3–5) underwent urinary clearance tests for TMAO, creatinine, uric acid, urea, and other TMAO precursors to calculate their fractional excretion (FE). Baseline characteristics of these patients are shown in Table S2. FE are shown in Table 4. The mean ± SD sodium fractional reabsorption for the three groups were 99.2% ± 0.6% evidencing that no patient exhibited any significant tubular disorder. Plasma TMAO, creatinine, and urea progressively increased with the loss of GFR. Due to tubular secretion, known to increase with the loss of renal function, clearance of creatinine was significantly higher than mGFR in all groups (*p* < 0.0001), with a mean ± SD value of FE of 137% ± 25%. Inversely, clearance of urea was significantly lower than mGFR (*p* < 0.0001), with a mean ± SD FE of 58% ± 19%. In contrast to urea or creatinine, TMAO FE was not significantly different from mGFR (mean, 105% ± 32%, *p* = 0.45). Clearances and FE of uric acid, choline, betaine and carnitine were also calculated (Table S3). Data show that renal excretion of uric acid, choline, betaine and carnitine were very limited with clearance medians [IQR] of 6 [4–11], 2 [1–4], 2 [1–6] and 2 [1–3] mL/min/1.73 m^2^ respectively, associated with low FE (9% [6–13%], 3% [2–6%], 2% [1–9%] and 3% [1–4%], respectively).

### 2.3. Hemodialysis Removal

**Figure 2:**
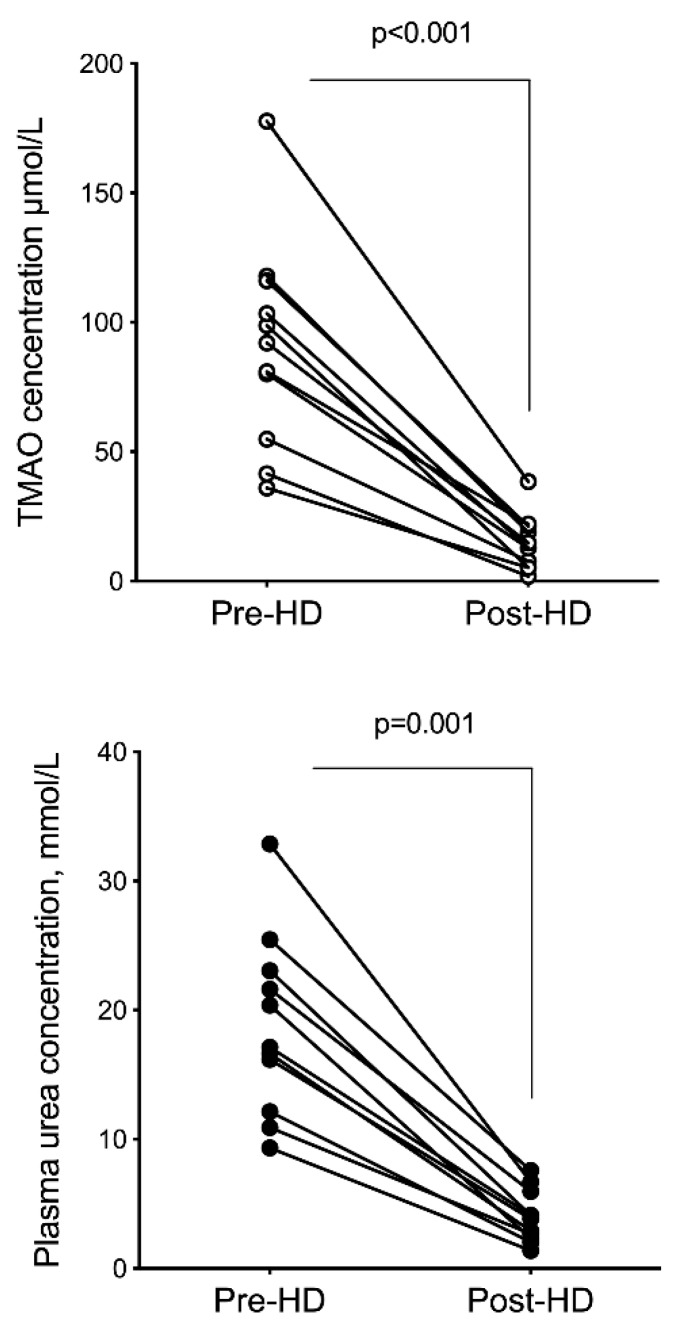
Plasma TMAO and urea levels before and after a hemodialysis session in 11 end-stage renal disease patients. TMAO and urea concentrations post hemodialysis were corrected for hemoconcentration as described in methods. Differences between pre- and post-dialysis concentrations were considered significant at the *p* < 0.05 level (Wilcoxon test for paired samples). Abbreviation: HD, hemodialysis.

Concentrations of TMAO, choline, betaine, and carnitine were measured before and immediately after a single hemodialysis session in 11 chronic hemodialysis patients, in order to calculate their fractional reduction (FR). All patients were treated for 4 h with hemodiafiltration. Clinical and biological characteristics of hemodialysis patients are shown in Table 1. Only one patient exhibited a significant residual diuresis. As expected, hemodialysis patients had higher levels of urea and TMAO than controls, with a greater increase in TMAO (28-fold) than urea (4-fold). Post-dialysis concentrations of TMAO, choline, betaine, and carnitine were significantly lower than pre-dialysis concentrations (*p* < 0.0001) (see Figure 2). TMAO FR was significantly higher than urea FR (84.9% ± 6.5% vs. 79.2% ± 5.7%, *p* = 0.04). Compared to urea FR, choline and betaine FR were significantly lower (*p* < 0.0001 and *p* = 0.002, respectively) whereas carnitine FR was not significantly different (*p* = 0.064) (Table 5).

## 3. Discussion

Identified mechanisms for target molecule elevation in CKD patients are either a decrease in renal excretion, an increase in endogenous production or both. Thus, TMAO levels could result from variations in production levels (including dietary precursor intake, TMA endogenous production from gut microbiota, TMA and TMAO intestinal absorption, and FMO3 enzymatic activity) or from its renal excretion. This study was therefore designed to assess the exact changes in TMAO values according to CKD stages as determined by gold standard measurements of GFR and its potential modifications during renal metabolism.

In the present study, we confirmed that TMAO is increased in CKD based on an inverse, although weak, correlation (*r^2^* = 0.388) between plasma TMAO and mGFR. Surprisingly, in all stages of CKD, we did not find any correlation between TMAO levels and its precursors, except a weak correlation with carnitine. Measured clearances of TMAO showed a complete renal excretion of TMAO by glomerular filtration with a steady FE of 105% during CKD, regardless of the stage. Unlike previous studies, we reported no evidence for effective contribution of tubular excretion or reabsorption during renal clearance of TMAO. In hemodialysis patients, we confirmed a greater increase in TMAO even though FR was similar to that of urea.

This study investigated for the first time TMAO levels in all stages of CKD using gold standard measures of GFR. Results of TMAO plasma concentrations in CKD were in good accordance with previous studies [14,15]. Median TMAO plasma levels found herein were similar to those published by Stubbs et al. in 2015 [14], even though their data was based on estimated GFR. Another study reported a negative correlation between TMAO and mGFR but both data sets were restricted to CKD stages 3 or more severe [15]. To the best of our knowledge, no study has performed urinary clearance measures of TMAO in CKD. Previously, Hai et al. published TMAO clearances in control subjects, reporting a higher clearance of TMAO compared to creatinine, suggesting an active secretion of TMAO [18]. Due to its low molecular weight (75 Da) and soluble non ionizable nature, with minimal protein-bound fraction, we consider TMAO to be entirely filtered through the glomerular basement membrane, which is the main known elimination route for TMAO. Our results showed a likely complete TMAO glomerular filtration with a steady FE of TMAO, regardless of CKD stages. TMAO clearance was similar to that of mGFR, higher than that of urea, and lower than that of creatinine, suggesting that TMAO is neither secreted as creatinine [20] nor reabsorbed as urea [21,22] by renal tubules. We however cannot exclude that the secretion rate exactly matched the reabsorption of TMAO. Furthermore, high fractional reabsorption of sodium indicates that study participants had no tubular impairment, which reinforces the validity of these data. However, the IQR of TMAO FE showed that some patients exhibited higher or lower clearances of TMAO compared to mGFR; especially in the control group where the median FE of TMAO was 103% [IQR 55–144]. This dispersion could be explained by several factors. First, urinary clearance of TMAO was not assessed at the exact same time points than mGFR, measured by inulin or iohexol clearance. Second, the variability of TMAO excretion could also be related to the amount of ingested TMA precursors. We unfortunately did not administer standardized meals to avoid this bias. Yet, we did not find a significant correlation between plasma levels of TMAO and total protein intake. Moreover, several studies in animal or cellular models have shown involvement of tubular transporters such as organic cation transporter 2 (OCT2) in TMAO cellular uptake and efflux [23,24,25]. This, however, has not been confirmed in humans unlike what has been previously described for creatinine [25]. We observed that elevation of TMAO is mostly due to a decrease in mGFR rather than tubular dysfunction. These results are in line with another recent study reporting that urinary TMAO/creatinine ratios (used as a surrogate of TMAO excretion rate) were not significantly different between control and CKD subjects [14].

Several studies showed a concomitant increase in TMAO and its precursors [8,11,15], suggesting that TMAO accumulation in CKD could result from an increased TMA gut production, related to higher TMA-containing nutrient intakes (choline, betaine, and carnitine) or to CKD-associated gut dysbiosis. Surprisingly, we only found a weak correlation between plasma TMAO and free carnitine and not with choline or betaine. We could not explain the negative association between carnitine and GFR. Rather than establishing a link between carnitine intakes and plasma TMAO levels, we hypothesize that a bias in patient selection, with possibly higher ingestion of carnitine, could explain this result. Moreover, a recent study demonstrated that carnitine may not be the main dietary precursor for TMAO as choline presents a higher TMA-generating potential [26]. The absence of link between TMAO and choline levels in our study cohort, leads us to conclude that variations in TMAO precursor intakes seem to be modestly involved in TMAO level. Taking into account the similar protein intake levels observed for control subjects, all CKD stages and HD patients in our cohort, the elevation of TMAO in CKD appears to be poorly impacted by the “TMA-containing food”. However, the positive correlation between TMA and mGFR suggests that the increased TMAO measured in CKD patients could partially be due to an increased metabolization of TMA by FMO3. A recent study supporting this hypothesis reported that a decrease in kidney function was associated with an enhancement of FMO activity in mice [27]. However, we did not understand the lack of correlation between TMA and TMAO in this population that could only be a consequence of unsaturated FMO3 enzymatic reaction. Of note, the volatile nature of TMA could also explain the lack of TMA increase observed in our study although it has been taken into consideration when measuring TMA. Of importance, these results remain limited by the peripheral blood measurement of the precursors which prevented us from considering the circulating levels of the precursors prior to hepatic metabolism.

The underlying cause for observed choline increase in CKD remains unclear. Its limited renal clearance indicates that this increase is not a consequence of kidney function. Increased values of choline could result from an additional metabolic pathway such as the generation of betaine and glycine during the urea cycle, as described by Hartiala et al. [28]. We could hypothesize that, in uremic conditions, urea cycle is downregulated, which would lead to lower choline contribution for urea generation and hence, an increase in choline circulating levels. Thus, as choline can cross back the intestinal barrier, it may be present in larger quantities in the digestive tract regardless of intake; and may enhance the gut-dependent TMA production, contributing to the increase of TMAO in CKD. We did not, however, observe betaine increase in CKD patients of our cohort, even though betaine is one of the metabolites involved in the choline to urea metabolic pathway.

In hemodialysis patients, we reported much higher plasma TMAO levels than in end-stage renal disease (ESRD) patients, as has been previously described [14,18,19]. Despite very close physicochemical properties between urea and TMAO, the elevation of the latter in HD patients was particularly high pre-dialysis but remained significantly higher post-dialysis when compared to controls. TMAO and urea FR in hemodialysis was high (roughly 80%), suggesting that an increased production of TMAO in ESRD could contribute to its accumulation. A previous study has already reported the mismatch between high rates of TMAO despite its efficient removal by hemodialysis. The authors hypothesized that it was related to a smaller volume of distribution than that of urea [18]. Unfortunately, we did not collect dialysates to calculate dialysis clearance of TMAO that would have enabled us to estimate the volume of distribution of TMAO.

The discrepancy between urea and TMAO increases in HD patients could also be explained by the different metabolic pathways involved. For instance, the mitochondrial cycle for urea which is impaired in CKD [29], could lead to a lesser accumulation of urea than TMAO in these patients. More studies are however needed to explore the TMAO production mechanism in CKD, especially in end-stage renal disease.

## 4. Conclusions

Elevation of TMAO levels in CKD is mostly related to the decrease of mGFR and may be in part due to an enhanced production of TMAO by FMO3 and/or by the promotion of TMAO production pathway rather than that of urea. This latter will have to be confirmed and should be the aim of futures studies. Renal elimination of TMAO seems to be poorly influenced by tubular secretion or reabsorption, suggesting that therapeutic target research to reduce TMAO levels should focus on its production mechanisms rather than its renal elimination.

## 5. Materials and Methods 

### 5.1. Subjects

From October 2012 to December 2016, 124 subjects were prospectively recruited from the Nephrology and Renal Functional Explorations Department in the Lyon teaching hospitals (Hôpital E. Herriot, Hospices Civils de Lyon, Lyon, France). Hemodialysis or CKD patients who were referred for GFR measurement were recruited to participate in the study. Renal transplant recipients and patients with systemic active inflammatory disease or known active malignant diseases were excluded. Controls subjects consisted of patients referred to the renal unit for kidney donation or for exploration of kidney stone and with normal kidney function. This research was approved by the local institutional review committee (reference number L16-88, Comité de Protection des Personnes -Recherche Biomédicale, CPP Lyon Sud-Est IV) and conducted in accordance with its ethical standards and the principles of the Declaration of Helsinki. All subjects involved in the research signed written informed consent forms prior to enrolment. 

### 5.2. Anthropometric Data

Body weight was measured in light clothing without shoes to the nearest 100 g on a digital scale, in order to determine dry weights for HD patients. Body mass index (BMI) was calculated as body weight divided by squared height. Body Surface Area (BSA) was assessed following the Dubois and Dubois BSA formula: BSA = (0.0071843 × total body weight (kilograms) ^0.425^ × height (centimeters) ^0.725^). 

### 5.3. Blood Sampling

After an overnight fast, blood samples were obtained by venipuncture, except for dialysis blood samples that were obtained immediately before and after dialysis from the arterial line of the mechanical bloodstream. Blood samples were centrifuged at 3500× *g* for 10 min to isolate plasma supernatant which was snap frozen in liquid nitrogen and stored at −20 °C until analysis.

### 5.4. Glomerular Filtration Rate Assessment

#### 5.4.1. Measured GFR (mGFR)

GFR was measured using gold standard methods, i.e., urinary clearance of inulin (mL/min per 1.73 m^2^) or iohexol clearance. Inulin clearance measurement (INUTEST 25%, Fresenius Kabi, Austria) was performed with a loading dose of 30 mg/kg injected over 10 min, followed by a maintenance inulin dose infusion of 40 mg/kg/h. Urine was collected every 30 min (3–4 collection periods of 30 min), and blood tests were performed between urine collection time points. Inulin clearance was calculated for each period to determine mean GFR for each subject, based on the following Equation (1):

(1) $$\mathrm{GFR}=\frac{\mathrm{Urinary}\;\mathrm{Inulin}\;\mathrm{concentration}\;\times \;\mathrm{Urinary}\;\mathrm{Volume}}{\mathrm{Plasma}\;\mathrm{Inulin}\;\mathrm{Concentration}}.$$

Measurements of plasma and urine inulin concentrations were performed using an enzymatic method. Iohexol clearance was performed with a single injection of 8 mL iohexol, 300 mg (Omnipaque, GE Healthcare SAS, Vélizy-Villacoublay, France) and by weighing the syringe before and after the injection. Blood collection was performed at 120, 180, and 240 min. Serum iohexol concentration was measured by High Performance Liquid Chromatography (HPLC). GFR was calculated using the following Equation (2):

(2) $$\mathrm{GFR}=\frac{\mathrm{Slope}\;\times \;\mathrm{Dose}}{\mathrm{Concentration}\;\mathrm{at}\;\mathrm{time}\;0}.$$

The concentration at time 0 was corrected using the Bröchner–Mortensen equation. 

#### 5.4.2. Estimated GFR (eGFR)

eGFR was estimated using the CKD EPI (Chronic Kidney. Disease - Epidemiology) formula [30] and CKD stages were determined according to K/DOQI guidelines [30].

### 5.5. Clearance and Fractional Excretion Calculations

Urinary clearance of creatinine, urea, uric acid, TMA, TMAO, choline, betaine, and carnitine was calculated by the following Equation (3):

(3) $$\mathrm{Clearance}\;\left(\frac{\mathrm{mL}}{\min}\right)\;=\frac{\mathrm{Urinary}\;\mathrm{concentration}\;\left(µ\mathrm{mol}/\mathrm{mL}\right)\;\times \;\mathrm{Urinary}\;\mathrm{rate}\;\left(\mathrm{mL}/\min\right)}{\mathrm{Plasma}\;\mathrm{concentration}\;\left(µ\mathrm{mol}/\mathrm{mL}\right)}.$$

Urine was collected for a precisely measured time to calculate urinary debits. All the clearance and GFR measurements were indexed to the BSA and expressed by 1.73 m^2^. Fractional Excretion (FE) was calculated using the following Equation (4):

(4) $$\mathrm{Fractional}\;\mathrm{Excretion}\;\left(\%\right)=\frac{\mathrm{Clearance}\;\left(\mathrm{mL}/\min\cdot 1.73m^{2}\right)\;}{\mathrm{mGFR}\;\left(\mathrm{mL}/\min\cdot 1.73m^{2}\right)\;}\times \;100.$$

### 5.6. Quantification of Methylamines

Trimethylamine N-oxide (TMAO), trimethylamine (TMA), betaine, choline, and carnitine concentrations were determined by liquid chromatography-tandem mass spectrometry (LC-MS/MS). All solvents used were LC-MS grade and purchased from Biosolve (Valkenswaard, Netherlands). Standard compounds were obtained from Sigma Aldrich (Saint-Quentin Fallavier, France). A pool of reference standard solutions was prepared and serially diluted in acetonitrile to obtain seven standard solutions ranging from 0.05 to 100 µmol/L. Urine samples were diluted 10-fold in distillated water prior to analysis. Exogenous internal standards (10 µL) diluted at 25 µmol/L in acetonitrile (^2^H_9_-choline, ^2^H_9_-carnitine, ^13^C_2_-betaine, [^13^C_3_,^15^N]-TMA and ^2^H_9_-TMAO) were added to 20 µL of standard solutions, plasma, and diluted urine samples. All samples were then treated with 75 µL of tert-butyl-bromoacetate (TMA derivatization) diluted at 50 mmol/L in acetonitrile and 10 µL of 70% ammonium hydroxide solution before mixing and incubation in the dark, at room temperature, for 30 min. Then, 50 µL of acetonitrile containing 1% formic acid were then added and samples were centrifuged for 10 min at 10,000× *g* (20 °C). Supernatants were then transferred to vials for LC-MS/MS analyses, performed on a Xevo^®^ TQD mass spectrometer with an electrospray interface and an Acquity H-Class^®^ UPLC^TM^ device (Waters Corporation, Milford, MA, USA). Samples (5 µL) were injected onto an HILIC-BEH column (1.7 µm, 2.1 × 100 mm, Waters Corporation) held at 35 °C. Compounds were separated using a linear gradient of mobile phase B (98% acetonitrile, 0.1% formic acid) in mobile phase A (10 mmol/L ammonium acetate, 0.1% formic acid) at a flow rate of 400 µL/min. Mobile phase A was kept constant for 1 min at 1%, linearly increased from 1% to 45% for 6.5 min, kept constant for 1 min, returned to the initial condition over 1 min, and kept constant for 1.5 min before the next injection. Targeted compounds were then detected by the mass spectrometer with the electrospray interface operating in the positive ion mode (capillary voltage, 1.5 kV; desolvatation gas (N_2_) flow and temperature, 650 L/h and 350 °C; source temperature, 150 °C). The multiple reaction monitoring mode was applied for MS/MS detection as detailed in Table S1. Chromatographic peak area ratios between unlabeled compounds and their respective internal standards constituted the detector responses. Standard solutions were used to plot calibration curves for quantification. The linearity was expressed by the mean *r²* which was greater than 0.997 for all compounds (linear regression, 1/x weighting, origin excluded). The intra- and inter-assay imprecisions of the analytical method were assessed throughout experiments in spiked samples with known concentrations (three experiments, five replicates per experiment for four spiked concentrations), and were below 11.4% for all compounds.

### 5.7. Other Biochemical Measurements

Plasma creatinine, urea, uric acid, bicarbonate, and protein concentrations were measured by standard laboratory methods in a certified laboratory. Cholesterol and triglyceride (TG) concentrations were measured using enzymatic test kits (Boehringer Mannheim GmbH, Mannheim, Germany). HDL cholesterol concentrations (HDL-C) were measured using a specific assay kit allowing apoB-containing lipoprotein precipitation (Cell Biolabs Inc., San Diego, CA, USA). LDL cholesterol concentrations (LDL-C) were then deducted using the Friedewald equation [31]. Patient protein intake was estimated from daily urinary excretion of urea for non-dialysis patients using the Equation 5:

(5) $$\mathrm{Protein}\;\mathrm{intake}\;\left(g/\mathrm{kg}.d\right)=\frac{\mathrm{Urinary}\;\mathrm{urea}\;\left(\mathrm{mmol}/\mathrm{day}\right)\;}{5\;\times \mathrm{Weight}\;\left(\mathrm{kg}\right)\;}\times \;100$$

For HD patients, protein intake was estimated by normalized protein catabolic rate (nPCR) with the following Equation 6 [32]:

nPCR = (pre dialysis urea × 2.801)/(25.8 + 1.15 _sp_Kt/V + 56.4/_sp_Kt/V) + 0.168 (6)

Post dialysis values of TMAO, choline, betaine, carnitine and urea were normalized according to the hemoconcentration (calculated by protein concentration ratio) of each patient.

### 5.8. Statistical Analyses

Data are expressed as mean ± standard deviation (SD) or as median [interquartile range, IQR] when variables were not normally distributed. Data were analyzed using Graphpad Prism version 8.2.1, (GraphPad softwares, La Jolla, CA, USA, 2019). Normality was tested using d’Agostino–Pearson test. Differences between groups were assessed with one-way ANOVA or a Kruskal–Wallis test, when variables were not normally distributed, completed by post-hoc multiple comparisons. Sex ratio and medications between groups were compared using Fisher exact test. Univariate analysis was performed using the Spearman rank correlation method. A *p* < 0.05 was considered as statistically significant in all analyses.

## Figures and Tables

**Table 1:** Baseline characteristics of Control subjects, Chronic Kidney Disease (CKD), and Hemodialysis patients.

Variable	Controls	CKD Patients			Hemodialysis	*p*-Value
		Stage 1–2	Stage 3a–3b	Stage 4–5		
Sex, male/female	13/5	26/23	9/22	12/3	7/4	0.0054
Age, y	41 [34–47] ^a^	46 [30–58] ^a^	64 [45–69] ^b,c^	50 [33–74] ^a,c^	62 [48–75] ^a,c^	0.0014
BMI, kg/m^2^	24.1 [22.7–25.7]	24.2 [20.6–26.0]	24.5 [21.6–27.2]	25.3 [21.0–30.9]	22.4 [21.1–27.5]	0.6427
Systolic BP, mmHg	127.6 ± 21.0	122.8 ± 17.3	134.2 ± 21.9	128.5 ± 22.3	115.5 ± 19.1	0.1538
Diastolic BP, mmHg	82.8 ± 13.5	77.8 ± 13.6	79.2 ± 13.3	79.7 ± 17.6	59.0 ± 2.8	0.2261
Creatinine, µmol/L	67.9 ± 14.6 ^a^	87.8 ± 18.0 ^a,b^	119.6 ± 36.6 ^b^	268.8 ± 113.2 ^c^	689.0 ± 149.8 ^d^	<0.0001
mGFR, mL/min. 1.73 m^2^	100 [93–107] ^a^	74 [66–83] ^b^	48 [41–54] ^c^	20 [14–24] ^c^	NA	<0.0001
Urea, mmol/L	4.4 [3.6–6.2] ^a^	5.9 [5.0–7.9] ^a,b^	9.3 [7.0–11.7] ^b,c^	21.5 [11.1–30.1] ^c^	16.1 [13.0–24.2] ^c^	<0.0001
UA/C, mg/mmol	0.8 [0.5–1.2] ^a^	1.2 [0.6–3.5] ^a,b^	2.5 [0.6–49.7] ^b,c^	38.3 [1.0–61.9] ^c^	NA	0.0001
Uric acid, mmol/L	252 [185–310] ^a^	275 [222–348] ^a^	317 [279–356] ^a,b^	555 [465–624] ^b^	ND	0.0008
Protein intake, g/kg/day	1.03 ± 0.34	0.96 ± 0.35	0.86 ± 0.23	0.81 ± 0.14	1.26 ± 0.47	0.3814
Bicarbonates, mmol/L	25.0 [24.0–27.0] ^a^	25.0 [23.8–27.0] ^a^	24.5 [23.3–26.0] ^a,b^	20.5 [18.0–32.8] ^a,b^	22.0 [20.0–24.0] ^b^	0.0122
Proteins, g/L	74 [71–78] ^a^	74 [71–78] ^a^	72 [66–74] ^a,b^	72 [66–76] ^a,b^	66 [65–71] ^b^	0.0015
Triglycerides, mmol/L	0.84 [0.66–1.11] ^a,c^	1.00 [0.77–1.40] ^a,b^	1.17 [1.00–1.54] ^b^	1.55 [0.81–1.95] ^b^	1.24 [1.04–1.53] ^b,c^	0.0021
Total cholesterol, mmol/L	4.82 [3.80–5.81] ^a,b^	5.22 [4.39–5.81] ^a^	4.84 [3.85–5.58] ^a,b^	3.99 [3.57–4.50] ^b^	3.26 [2.79–4.00] ^b^	0.0004
HDL-cholesterol, mmol/L	1.03 [0.85–1.47]	1.16 [1.02–1.43]	1.11 [0.98–1.29]	0.95 [0.82–1.27]	1.14 [0.94–1.21]	0.1569
LDL-cholesterol, mmol/L	3.42 [2.36–4.06] ^a^	3.45 [2.73–3.97] ^a^	3.00 [2.10–3.49] ^a,b^	2.45 [1.71–2.81] ^b^	1.82 [1.22–2.16] ^b^	<0.0001
Lipid Lowering treatments, %	0.0	2.4	30.0	40.0	81.8	<0.0001

Data are expressed as means ± standard deviation and compared with one-way ANOVA when the values passed normality test or are expressed as medians [interquartile range] and compared with a Kruskal–Wallis test when values did not pass normality test. BP: blood pressure; BMI: body mass index; CKD: chronic kidney disease; mGFR: measured glomerular filtration rate; NA: non-applicable; ND: not determined; UA/C: urinary albumin/creatinine ratio. Different letters indicate a significant difference between groups (*p* < 0.05).

**Table 2:** Univariate analysis of clinical and biological parameters with Trimethylamine-N-oxide (TMAO) plasma concentrations (µmol/L).

Variable	*r_s_*	95% CI	
Age, y	0.30	0.12 to 0.47	**
BMI, kg/m^2^	0.17	−0.02 to 0.35	ns
UA/C, mg/mmol	0.24	0.06 to 0.42	**
Uric Acid, µM	0.31	0.10 to 0.50	**
Bicarbonates, mM	−0.22	−0.43 to 0.01	*
Protein intake, g/kg/day	0.06	−0.21 to 0.31	ns
Triglycerides	0.17	−0.02 to 0.35	ns
HDL-cholesterol, mmol/L	−0.10	−0.28 to 0.09	ns
LDL-cholesterol, mmol/L	−0.16	−0.34 to 0.03	ns
TMA, µmol/L	−0.14	−0.32 to 0.06	ns
Choline, µmol/L	0.18	−0.01 to 0.36	ns
Betain, µmol/L	−0.05	−0.23 to 0.15	ns
Carnitine, µmol/L	0.20	0.01 to 0.38	*

Univariate correlations were performed using two-tailed Spearman’s test with 95% confidence interval for *n* = 61 to 113, according to the available data. * *p* < 0.05 ** *p* < 0.005. Abbreviations: A/CU: Urinary albumin/creatinine; BMI: body mass index; ns: non-significant; TMAO: trimethylamine-N-oxide.

**Table 3:** Plasma concentrations of TMAO precursors.

Analyte	Controls	CKD Patients			Hemodialysis	*p*-Value
		Stage 1–2	Stage 3a–3b	Stage 4–5		
N	18	49	31	15	11	
Choline, µmol/L	1.10 ± 0.22 ^a^	1.03 ± 0.21 ^a^	1.11 ± 0.27 ^a^	1.31 ± 0.28 ^a^	3.32 ± 1.02 ^b^	<0.0001
Betain, µmol/L	40.15 [29.98–56.25]	29.90 [31.33–38.50]	32.20 [22.40–39.20]	33.40 [21.30–37.40]	40.13 [24.98–58.43]	0.0516
Carnitine, µmol/L	52.83 ± 18.55 ^a^	49.71 ± 13.07 ^a^	57.58 ± 16.91 ^a^	79.44 ± 31.62 ^b^	21.11 ± 7.73 ^c^	<0.0001
TMA, µmol/L	0.28 [0.26–0.32] ^a^	0.27 [0.25–0.31] ^a^	0.21 [0.18–0.28] ^b^	0.23 [0.21–0.28] ^a,b^	ND	<0.0001

Data are expressed as means ± standard deviation and compared with one-way ANOVA test when values passed normality test or are expressed as medians [interquartile range] and compared with a Kruskal–Wallis test when values did not pass normality test. CKD: chronic kidney disease; TMA: trimethylamine. Different letters indicate a significant difference between groups (*p* < 0.05).

**Table 4:** Measured glomerular filtration rate (mGFR) and renal excretion of TMAO, creatinine, and urea.

Group	Parameter	mGFR, mL/min/1.73 m^2^	FR _Na_ %	pTMAO µmol/L	Cl _TMAO_ mL/min/1.73 m^2^	FE _TMAO_ %	pCreat µmol/L	Cl _Creatinine_ mL/min/1.73m^2^	FE _Creat_ %	pUrea mmol/L	Cl _Urea_ mL/min/1.73m^2^	FE _Urea_ %
Controls	median	98	99.4	2.4	109	103	72	125	127	4.8	53	51
*n* = 5	IQR	[91–105	[98.8–99.6]	[2.1–30.7]	[50–145]	[55–144]	[68–82]	[104–148]	[114–141]	[3.7–5.5]	[39–77]	[42–76]
CKD stages 1–2	median	73	99.4	3.5	71	106	94	94	133	5.5	38	56
*n* = 19	IQR	[67–79]	[99.0–99.7]	[2.4–4.9]	[57–89]	[86–118]	[84–109]	[81–116]	[120–146]	[3.8–6.4]	[31–52]	[46–66]
CKD stages 3–5	median	51	99.1	9.2	55	108	123	73	153	8.3	30	61
*n* = 8	IQR	[46–55]	[97.9–99.5]	[5.4–14.0]	[43–67]	[98–130]	[96–151]	[61–84]	[132–161]	[6.3–10.7]	[18–44]	[51–82]
*p*-value		<0.0001	0.401	0.134	0.048	0.899	0.001	0.002	0.121	0.005	0.051	0.721

Data are expressed as medians [interquartile range] and compared with a Kruskal–Wallis test. Abbreviations: CKD: chronic kidney disease; Cl: clearance; F: fractional excretion; FR: fractional reabsorption; IQR: interquartile range; mGFR: measured glomerular filtration rate; p: plasma; TMAO: trimethylamine-N-oxide.

**Table 5:** Metabolites hemodialysis removal.

Analyte	Pre-Dialysis	Post-Dialysis	FR	*p*
TMAO, µmol/L	90.84 ± 40.11	14.65 ± 10.39	84.91 ± 6.49	<0.0001
Choline, µmol/L	3.32 ± 1.02	1.76 ± 0.64	46.72 ± 14.30	<0.0001
Betaine, µmol/L	42.68 ± 17.34	16.34 ± 7.62	61.26 ± 10.53	<0.0001
Carnitine, µmol/L	21.11 ± 7.73	4.45 ± 1.50	77.89 ± 7.29	<0.0001
Urea, mmol/L	18.7 ± 6.93	3.97 ± 2.01	79.21 ± 5.66	<0.0001

Data are expressed as means ± SEM and compared with paired *t*-test with significant *p* < 0.05. FR: fractional reduction; TMAO: trimethylamine-N-oxide.

## Supplementary Materials

The following are available online at https://www.mdpi.com/2072-6651/11/11/635/s1. Table S1: Multiple Reaction Monitoring (MRM) parameters used for LC-MS/MS analysis. Table S2: Baseline characteristics of Control subjects and CKD patients who performed TMAO clearance. Table S3: mGFR and renal excretion of uric acid, choline, betaine, and carnitine. Figure S1: Typical calibration curve of TMAO in LC-MS/MS. Figure S2: Representative LC-MS/MS chromatograms of TMAO in pasma (A) and urine (B). The deuterated standard (TMAO D9) is presented in green (lower panel). Figure S3: Representative LC-MS/MS chromatograms of TMAO at the lower limit of quantification (LLOQ). The deuterated standard (TMAO D9) is presented in red (lower panel).
