# Supplementary material for: Elevation of Trimethylamine-N-Oxide in Chronic Kidney Disease: Contribution of Decreased Glomerular Filtration Rate
Source: Toxins (Basel). 2019 Nov 1;11(11):635. doi: 10.3390/toxins11110635 (PMC6891811; doi:10.3390/toxins11110635)
Supplement: Supplementary file 1 [file toxins-11-00635-s001.pdf]

Supplementary Materials: Elevation of Trimethylamine-N-Oxide in Chronic Kidney Disease: Contribution of Decreased Glomerular Filtration Rate

Caroline C. Pelletier, Mikael Croyal, Lavinia Ene, Audrey Aguesse, Stephanie Billon-Crossouard, Michel Krempf, Sandrine Lemoine, Fitsum Guebre-Egziabher, Laurent Juillard and Christophe O. Soulage

Table S1. Multiple Reaction Monitoring (MRM) parameters used for LC-MS/MS analysis.

Compounds	MRM (*m*/*z*)	Cone Voltage (V)	Collision Energy (eV)
TMAO	75.9 → 58.9	20	11
$^{2}H_{9}$-TMAO	85.0 → 68.0	20	11
Derivatized TMA	174.1 → 118.0	25	10
Derivatized [$^{13}C_{3}$,^{15}N]-TMA	178.1 → 122.0	25	10
Betaine	118.1 → 58.1	40	22
$^{13}C_{2}$-betaine	120.1 → 58.1	40	22
Choline	104.1 → 60.1	40	15
$^{2}H_{9}$-choline	113.2 → 69.1	40	15
Carnitine	162.1 → 103.0	25	14
$^{2}H_{9}$-carnitine	171.1 → 69.1	25	14

Table S2. Baseline characteristics of Control subjects and CKD patients who performed TMAO clearance.

Variable	Controls	CKD Patients		*p*-Value
		Stage 1–2	Stage 3–5	
N	5	19	8	
Sex, male/female	2/3	12/7	3/5	0.387
Age, y	44 [34–50]	37 [23–57]	57 [23–69]	0.508
BMI, kg/m^2	22.4 [19.8–27.3]	23.7 [21.7–26.0]	23.5 [21.6–29.1]	0.852
Systolic Blood Pressure, mmHg	109 [96–146]	113 [105–130]	117 [101–135]	0.782
Diastolic Blood Pressure, mmHg	73 [61–89]	72 [64–81]	68 [59–77]	0.735
Heart Rate, bpm	74 [61–79]	62 [57–70]	66 [57–75]	0.434
Creatinine, µmol/L	72 [62–82] [a]	94 [84–109] [a,b]	123 [96–151] [b]	0.001
mGFR,mL/min/1,73m^2	98 [91–105] [a]	73 [67–79] [b]	51 [46–55] [c]	<0.0001
Urea, mmol/L	4.8 [3.7–5.5] [a]	5.5 [3.8–6.4] [a]	8.3 [6.3–10.7] [b]	0.005
UA/C, mg/mmol	0.4 [0.4–1.1]	0.9 [0.4–1.7]	3.5 [0.8–59.0]	0.101
Uric acid, mmol/L	252 [217–298]	319 [261–449]	390 [322–635]	0.063
Proteins intake, g/kg/day	1.02 [0.87–1.40]	0.82 [0.70–0.82]	0.97 [0.77–1.08]	0.218
Bicarbonates, mmol/L	24 [22–25]	25 [24–26]	26 [23–34]	0.277
Proteins, g/L	71 [70–74]	72 [70–75]	69.5 [65.3–72.8]	0.218
Triglycerides, mmol/L	0.94 [0.81–1.35] [a,b]	0.92 [0.83–1.88] [a]	2.12 [1.54–3.18] [b]	0.025
Total cholesterol, mmol/L	4.11 [3.92–4.79]	5.42 [4.19–5.90]	4.83 [3.76–5.42]	0.205
HDL-cholesterol, mmol/L	1.08 [0.98–1.97]	1.18 [1.07–1.68]	0.92 [0.79–1.31]	0.221
LDL-cholesterol, mmol/L	2.76 [2.24–2.90]	3.49 [2.40–4.08]	2.90 [1.99–3.50]	0.244
Lipid Lowering treatments, %	0.0	5.3	55.5	0.076

Data are expressed as medians [interquartile range] and compared with a Kruskal-Wallis test. Categorical data were compared using Fisher's exact test. BMI: body mass index; CKD: chronic kidney disease; CRP: C-reactive protein; CV: cardiovascular; mGFR: measured glomerular filtration rate; RAA: renin angiotensin aldosterone; UA/C: urinary albumin/creatinine ratio. Different letters indicate a significant difference between groups ($p < 0.05$).

Table S3. mGFR and renal excretion of uric acid, choline, betaine and carnitine.

Variable	Parameter	mGFR, mL/min/1.73 m²	FR_{Na} %	pUric acid µmol/L	$Cl_{Uric acid}$ mL/min/1.73m²	$FE_{Uric acid}$ %	pCholine µmol/L	$Cl_{Choline}$ mL/min/1.73 m²	$FE_{Choline}$ %
Controls	median	98	99.4	252	9	9	1.2	3	3
n = 5	IQR	[91–105]	[98.8–99.6]	[217–298]	[7–12]	[8–13]	[1.0–1.4]	[1–8]	[2–8]
CKD stages 1–2	median	73	99.4	319	6	8	1.3	2	3
n=19	IQR	[67–79]	[99.0–99.7]	[261–449]	[4–11]	[6–15]	[1.2–1.5]	[1–4]	[2–5]
CKD stages 3–5	median	51	99.1	390	5	9	1.9	3	7
n=8	IQR	[46–55]	[97.9–99.5]	[322–635]	[3–7]	[7–15]	[1.5–2.1]	[2–5]	[3–10]
p-value		<0.0001	0.401	0.063	0.117	0.262	0.005	0.796	0.172
Controls	median	98	99.4	29.9	2	2	40.2	2	1
n=5	IQR	[91–105]	[98.8–99.6]	[14.8–46.3]	[2–4]	[2–4]	[30.8–47.3]	[1–3]	[1–3]
CKD stages 1–2	median	73	99.4	32.8	1	2	40.8	2	3
n = 19	IQR	[67–79]	[99.0–99.7]	[27.3–38.4]	[1–6]	[1–8]	[35.7–44.3]	[1–3]	[1–4]
CKD stages 3–5	median	51	99.1	29.0	4	8	60.3	2	4.0
n = 8	IQR	[46–55]	[97.9–99.5]	[15.4–36.3]	[1–12]	[3–23]	[36.7–67.8]	[1–3]	[2.3–5.0]
p-value		<0.001	0.401	0.365	0.543	0.079	0.174	0.983	0.145

Data are expressed as medians [interquartile range] and compared with a Kruskal-Wallis test. Abbreviations: CKD: chronic kidney disease; Cl: clearance; FE: fraction of excretion; FR: fraction of reabsorption; IQR: interquartile range; mGFR: measured glomerular filtration rate; p: plasma; TMAO: trimethylamine-N-oxide.

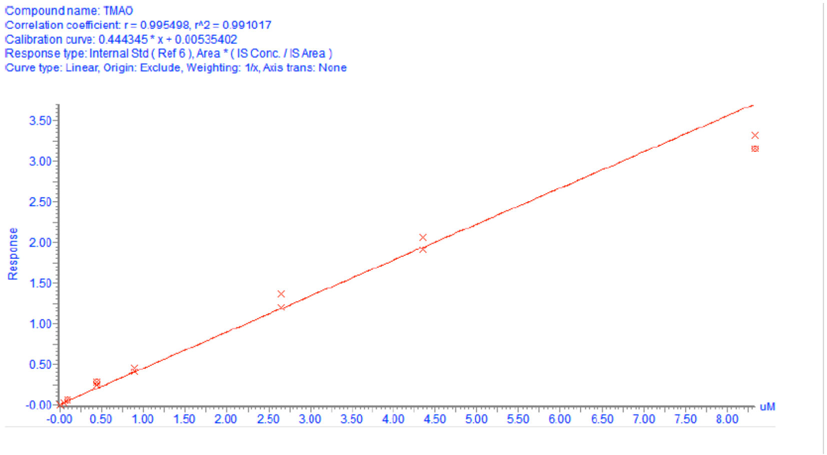

Figure S1. Typical calibration curve of TMAO in LC-MS/MS.

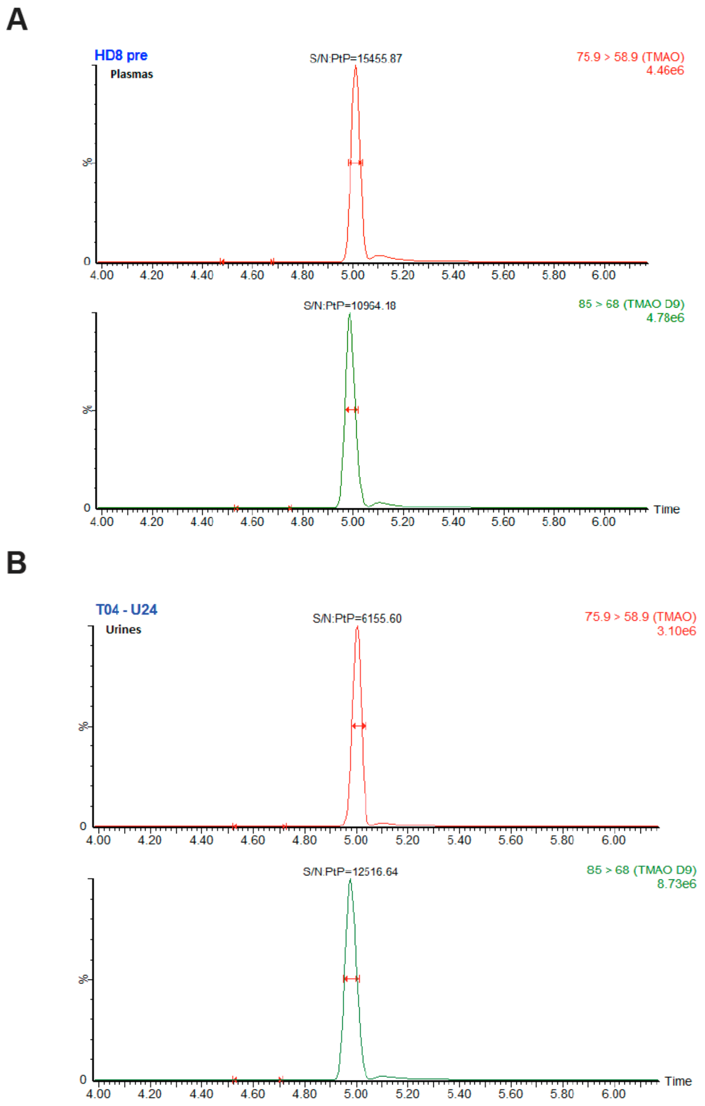

Figure S2. Representative LC-MS/MS chromatograms of TMAO in pasma (**A**) and urine (**B**). The deuterated standard (TMAO D9) is presented in green (lower panel).

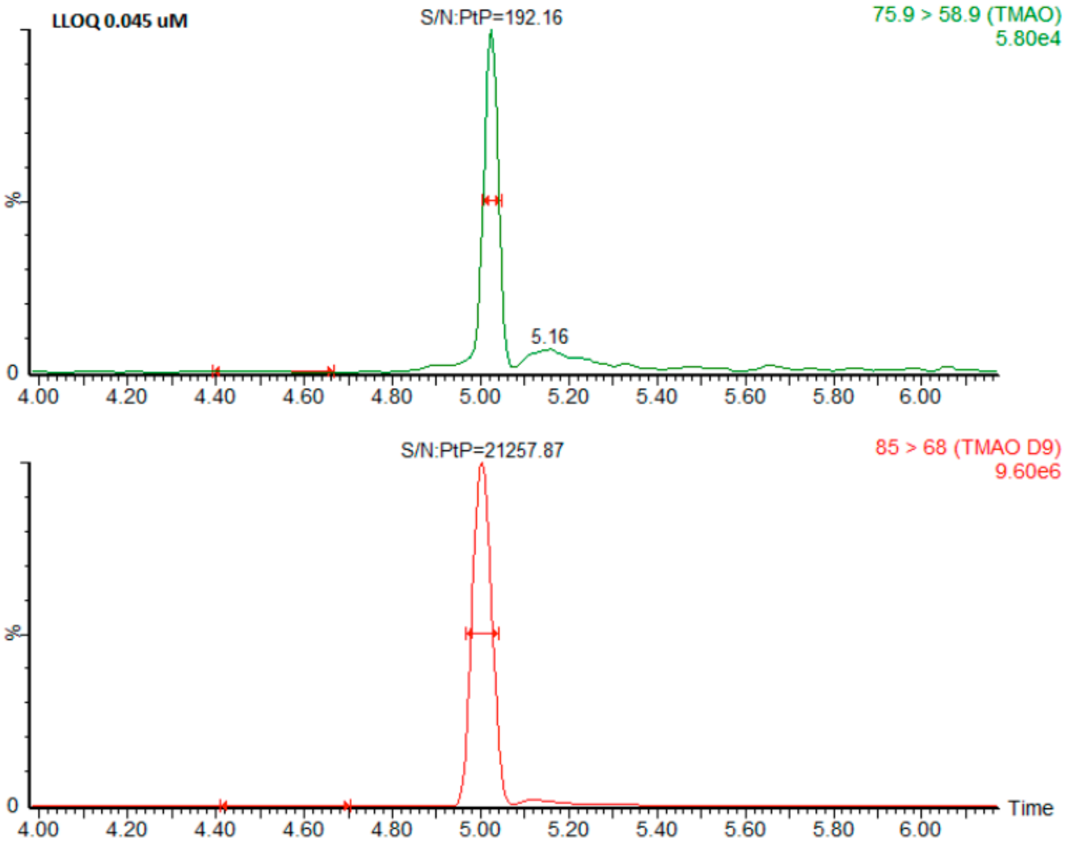

Figure S3. Representative LC-MS/MS chromatograms of TMAO at the lower limit of quantification (LLOQ). The deuterated standard (TMAO D9) is presented in red (lower panel).
